# Microbial diversity characteristics and differential analysis in green turtle nests in the Xisha Islands, South China Sea

**DOI:** 10.1128/aem.00527-25

**Published:** 2025-07-23

**Authors:** Xiaoyu An, Ting Zhang, Xin Niu, Yupei Li, Yangfei Yu, Yongkang Jiang, Jichao Wang, Haitao Shi, Li Ding, Liu Lin

**Affiliations:** 1 Ministry of Education Key Laboratory for Ecology of Tropical Islands, Key Laboratory of Tropical Animal and Plant Ecology of Hainan Province, College of Life Sciences, Hainan Normal University 117783, Haikou, China; 2 Hainan Sansha Provincial Observation and Research Station of Sea Turtle Ecology, Sansha, China; 3 Marine Protected Area Administration of Sansha City, Sansha, China; University of Delaware, Lewes, Delaware, USA

**Keywords:** *Chelonia mydas*, nest microorganisms, 16S rRNA, Xisha Islands

## Abstract

Sea turtles are renowned “living fossils” and ideal flagship marine conservation species. Sea turtles display high fidelity to their nesting grounds, and the quality of nesting grounds is related to the survival of sea turtles, in which microbial diversity, abundance, and potential pathogens are important indicators for assessing the quality of nesting grounds. The Xisha Islands are the largest nesting grounds for green turtles (*Chelonia mydas*) in China. Protecting the security and sustainability of the nesting grounds is of significant importance. This study compared and analyzed the bacterial community composition and characteristics of the nests of green turtles using high-throughput Illumina sequencing technology. The results showed that the sand of nests before hatching showed higher bacterial diversity and richness than that after hatching (*P* < 0.001). Additionally, the bacterial diversity and richness of the hatched eggshells were significantly higher than those of unhatched egg contents and the gastrointestinal tracts of deceased hatchlings (*P* < 0.001). The relative abundance of *Bacillus*, *Streptomyces* in the sand of nests after hatching was significantly decreased compared to that in the sand of nests before hatching (*P* < 0.05); however, the relative abundance of *Flavobacterium*, *Brevundimonas*, *Acinetobacter* increased significantly (*P* < 0.001). Most of the bacteria identified in our study are opportunistic pathogens that cause infections in plants and animals. The unhatched egg contents had a larger amount of opportunistic pathogens than hatched eggshells and the gastrointestinal tracts of deceased hatchlings. The presence and long-term accumulation of these pathogens may disrupt the ecological balance of the microbial community in nesting grounds, increase the susceptibility of sea turtle eggs, and pose a threat to the hatching and hatchling survival of sea turtle eggs, thereby posing a certain level of safety risk. The outcomes of this study could offer scientific support for the management and restoration of the nesting grounds in the Xisha Islands.

**IMPORTANCE:**

Many studies have shown that microorganisms influence the reproduction and hatching of sea turtles. The quality of nesting grounds is critically important for the successful reproduction of sea turtles, determining success in egg laying and influencing factors that affect the health of sea turtle populations. The Xisha Islands are the largest nesting grounds for green turtles in China. Regular and preventive monitoring of the microbial community composition in the sand and nests of nesting sites can contribute to the management and conservation of the nests.

## INTRODUCTION

Currently, sea turtles are recognized as one of the most endangered animals in the world, as only two families, six genera, and seven species of sea turtles are left. Sea turtles are apex consumers in marine food chains and are considered flagship species in marine ecosystems. They also serve as crucial indicators for marine environmental monitoring, significantly promoting marine material cycling and energy flow (1, 2).

Destruction and degradation of nesting grounds can lead to reproductive failure in sea turtles; therefore, monitoring and protecting sea turtle nesting grounds are central to global sea turtle conservation efforts (3). Sea turtles spend their entire lives in the ocean, with female sea turtles coming ashore only during the breeding season to lay eggs. Additionally, female sea turtles display high fidelity to their nesting grounds, undertaking long migrations back to the beaches where they were born to reproduce each breeding season (4). However, sea turtles have no brooding and rearing behavior; the eggs naturally incubate within the nests, and after 40–60 days, the hatchlings emerge and enter the sea to live independently. Furthermore, the quality of nesting grounds is critically important for the successful reproduction of sea turtles, determining success in egg laying and influencing factors such as hatching success rate, the survival rate of hatchlings, and other factors that affect the health of sea turtle populations (5).

Recently, many studies have shown that microorganisms influence the reproduction and hatching of sea turtles. Excessive microorganisms in sea turtle nests can adversely affect the hatching of sea turtle eggs (6). In early studies, bacterial pathogens constituted the longest list of infection hazards to sea turtles, the primary cause of disease in both captive and wild sea turtles is due to bacterial infections (7, 8). *Citrobacter freundii* has been proven to be a particularly notorious pathogen found in captive sea turtles (9). Additionally, among fungi, the genus *Fusarium* competes with embryos for calcium content in the eggshell and uses embryonic tissue as a nutrient source, directly leading to embryo death (10). In addition, microorganisms within nests can purify the microenvironment of the nests by producing antimicrobial compounds, such as *Streptomyces* and *Micromonospora*, which may possess antifungal activity capable of inhibiting the proliferation of harmful molds, thereby protecting the development of turtle eggs (11).

The Nancite nesting grounds in Costa Rica are globally renowned nesting grounds for the olive ridley sea turtle (*Lepidochelys olivacea*). However, monitoring has revealed a continuous decline in female sea turtles over 36 years, primarily due to contamination at the nesting grounds and poor microenvironment conditions in sea turtle nests, which lead to embryo mortality (12). High microbial loads may lead to a decline in turtle hatch rates (13).

In conserving the nesting grounds of international sea turtles, microbial diversity, abundance, and potential pathogens are important indicators for assessing the quality of nesting grounds. The degree of variation for such indicators can reflect the overall environmental conditions and quality status of the nesting grounds (14, 15). Moreover, the development of reptile eggs is closely associated with the microbial community in the soil. The embryos of green turtles that rely on nesting to reproduce are exposed to sand at a depth of approximately 60 cm in the nesting grounds for most of their developmental period, and throughout this period, the microbes in the environment accompany the turtle eggs during their entire development outside the maternal body. Therefore, regular and preventive monitoring of the microbial community composition in the sand and nests of nesting sites can contribute to the management and conservation of the nests (15).

The Xisha Islands are the largest nesting grounds for green turtles (*Chelonia mydas*) in China, with more than 200 nests recorded every year. Recent genetic studies have identified a new geographic population of green turtles breeding in the Xisha Islands of China, which primarily inhabit the South China Sea region and possess significant research and conservation value; however, the genetic diversity of the sea turtle population in Xisha is low and at risk of further decline, necessitating stricter conservation measures (16, 17). We compared the bacterial community characteristics of sand in the nests of green turtles before and after hatching and that in hatched eggshells, unhatched egg contents, and gastrointestinal tracts of deceased hatchlings using high-throughput sequencing technology, revealed the diversity and abundance of bacteria within the nests of green turtles, and provided critical scientific evidence for the planning, environmental management, and conservation-restoration of the nesting grounds of green turtles in the Xisha Islands.

## MATERIALS AND METHODS

### Sample collection

**Fig 1:**
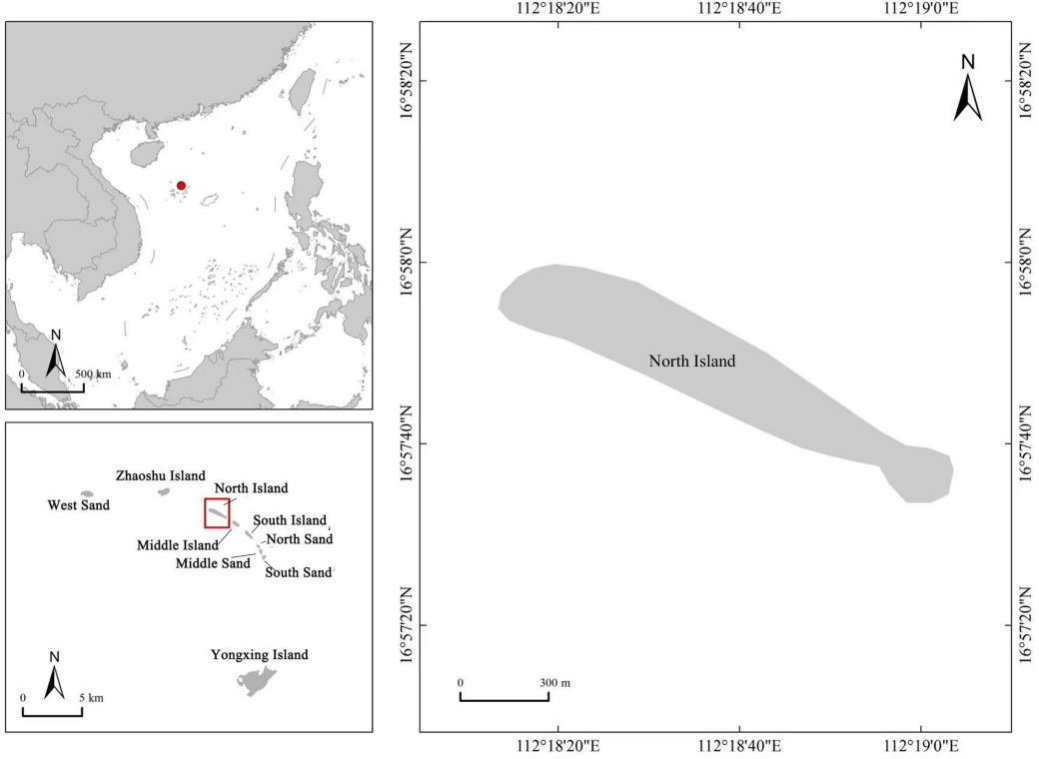
Study area map.

This study was conducted at the nesting grounds of green turtles on the North Island in the Xisha Islands of China (Fig. 1). Regular patrols were conducted during the breeding season when female green turtles came ashore to lay eggs. We randomly collected soil samples from different positions within the nest after the female turtles had finished digging the nest. Furthermore, the location and time of sand collection from nests after the female sea turtles had completed nesting were marked and recorded, respectively. We immediately collected sand from around the eggshells within the nest and samples of hatched eggshells, unhatched egg contents, and gastrointestinal tracts of deceased hatchlings 72 hours after the hatchlings had emerged from the nest. The unhatched eggs were opened using a sterile scalpel, and the contents were collected with a sterile pipette; deceased hatchlings were dissected with sterile scissors, and the gastrointestinal tracts were transferred into sterile centrifuge tubes using sterile tweezers (18, 19). Six or more normal hatched eggshells were randomly selected from each nest. In contrast, other samples were collected based on the specific conditions of each nest. All samples were homogenized and placed into 50 mL sterile centrifuge tubes, which were stored at −20°C until DNA extraction. The sand samples in this study were obtained from 12 green turtle nests (24 samples), the samples of hatched eggshells, unhatched egg contents, and gastrointestinal tracts of deceased hatchlings were obtained from eight nests of green turtles (24 samples).

### DNA extraction

Microbial genomic DNA was extracted using the K3115-S Soil and Fecal Genomic DNA Mini Kit (Guangzhou, China). DNA extraction quality was evaluated by agarose gel electrophoresis after extracting total microbial DNA from samples following the extraction kit instructions, and DNA concentration was quantified using the NanoDrop 2000 ND-2000 Spectrophotometer (Thermo Scientific, Wilmington, DE, USA).

### PCR amplification and sequencing

The V3–V4 hypervariable regions of the bacterial 16S rRNA gene were amplified using the bacterial primers 341F (5′-CCTACGGGNGGCWGCAG-3′) and 805R (5′-GACTACHVGGGTATCTAATCC-3′) (20). The PCR reaction mixture included Phusion Hot Start Flex 2× Master Mix (12.5 µL), Forward Primer (2.5 µL), Reverse Primer (2.5 µL), and Template DNA (50 ng). Finally, the volume of the PCR reaction mixture was adjusted to 25 µL with ddH_2_O. The PCR reaction conditions were as follows: initial denaturation at 98°C for 30 seconds, denaturation at 98°C for 10 seconds, annealing at 54°C for 30 seconds and 35 cycles, extension at 72°C for 45 seconds, and stable extension at 72°C for 10 minutes. Finally, the amplified samples were stored at 4°C. PCR products were purified using AMPure XT beads (Beckman Coulter Genomics, Danvers, MA, USA) and quantified with a Qubit 3.0 Fluorometer (Thermo Fisher Scientific, USA) (Thermo Fisher Scientific, USA). The amplification products were detected by 2% agarose gel electrophoresis, and recovery was performed using the AMPure XT beads purification kit. Sequencing was performed by Guangzhou IGE Biotechnology Co., Ltd. (Guangzhou, China) using the Illumina NovaSeq 6000 platform for paired-end sequencing with read lengths of 2 × 150 bp.

### Data analysis

The paired-end reads obtained from sequencing were initially assembled based on overlap relationships. Concurrently, sequence quality control and filtering were performed. The samples were differentiated to obtain the effective sequence according to the barcode and primer sequences at both ends of the sequence, and the direction of the sequence was corrected. Bases with quality scores below 20 at the end of reads were filtered out, and reads shorter than 50 bp after quality control were discarded. Additionally, reads containing “N” bases were excluded. A maximum mismatch ratio of 0.2 was allowed in the overlapping region of merged sequences after adjusting the sequence orientation. The barcode permitted zero mismatches, whereas the primer allowed a maximum of two. OTU taxonomic classification was performed using the RDP Classifier (http://rdp.cme.msu.edu/, version 2.11) with alignment against the Silva database (Release 138, http://www.arb-silva.de), with a confidence threshold of 70%, and the community composition of each sample was analyzed at various taxonomic levels.

### Statistical analysis

The data were processed and analyzed on the Majorbio Cloud platform. Species diversity indices were analyzed using Mothur (v.1.30.2, University of Michigan, Ann Arbor, MI, USA), and β-diversity distance matrices were calculated based on the Bray-Curtis distance algorithm. Linear discriminant analysis effect size (LEfSe) analysis was conducted for differential species identification at multiple taxonomic levels, from phylum to genus, to assess the differences in species between the groups. Statistical analysis was conducted using the SPSS 18.0 software package. The data do not conform to the normal distribution; thus, non-parametric data were analyzed using the Wilcoxon rank-sum test and Kruskal–Wallis H test to assess inter-group differences, with significance set at *P* < 0.05. The plots were generated using the plotting tools provided by Origin 2023 software and the Majorbio Cloud platform.

## RESULTS

### Analysis of 16S rRNA gene sequencing data

**Fig 2:**
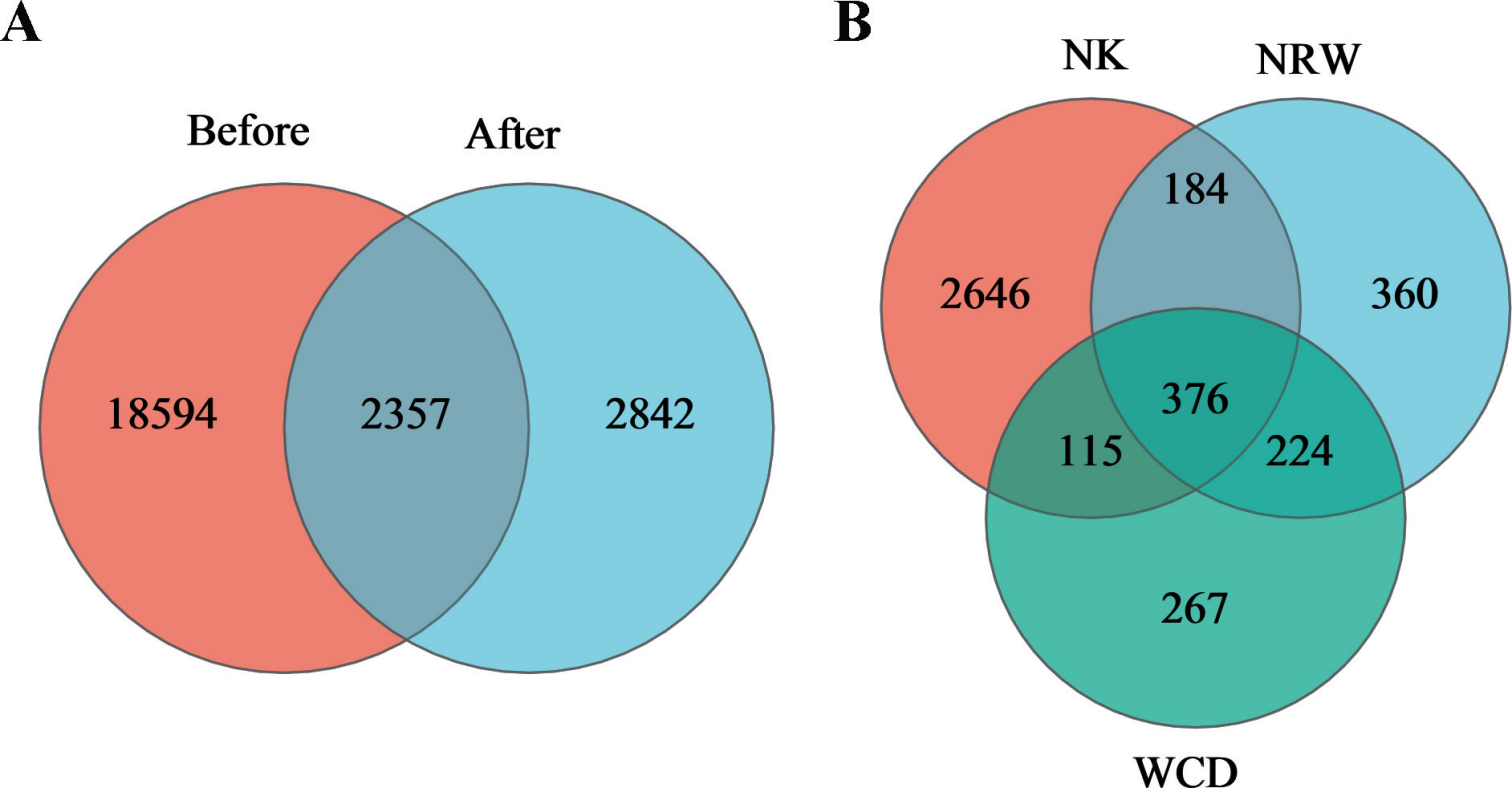
Venn diagram of OTU distribution of bacteria in the sand of nests before and after hatching (**A**). Venn diagram of OTU distribution of bacteria in hatched eggshells (NK), unhatched egg contents (NRW), and gastrointestinal tracts of deceased hatchlings (WCD) (**B**).

A total of 3,418,017 quality filter sequences were obtained after the detection of 48 samples (Table 1). The average sequence base length for all samples was 468 bp. Clustered at a 97% similarity threshold, a total of 26,150 OTUs were obtained from the sand of nests before and after hatching (Fig. 2A); of these, a total of 18,594 and 2,842 unique OTUs in the sand of nests before and after hatching, respectively, were observed, and 2,357 OTUs were shared between both categories. A total of 5,447 OTU were obtained from hatched eggshells, unhatched egg contents, and gastrointestinal tracts of deceased hatchlings (Fig. 2B); of these, 2,646, 360, and 267 unique OTU in hatched eggshells, unhatched egg contents, and gastrointestinal tracts of deceased hatchlings, respectively, were observed, and 376 OTU were shared among all three categories.

**TABLE 1:** Results of high-throughput sequencing of bacterial communities in different groups

Sample grouping	Number of quality filter sequences	Average base length (bp)	Number of OTUs
Sand of nests before hatching (before)	1,340,591	457	20,951
Sand of nests after hatching (after)	897,314	466	5,199
Hatched eggshells (NK)	448,479	468	3,321
Unhatched egg contents (NRW)	322,024	475	1,144
Gastrointestinal tracts of deceased hatchlings (WCD)	409,609	475	982

### Comparative analysis of microorganisms in the sand of nests before and after hatching

#### Analysis of alpha and beta diversities

**Fig 3:**
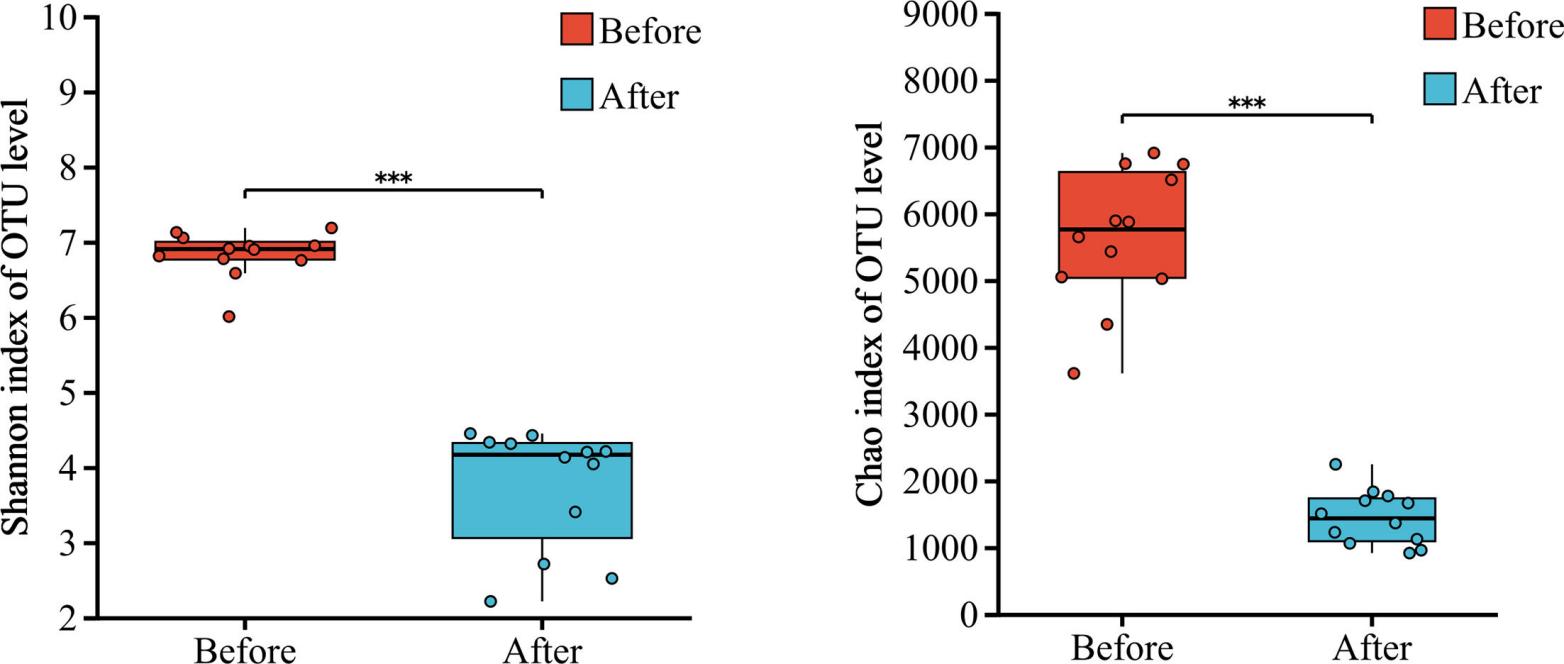
Alpha diversity analysis of bacterial communities in the sand of nests before and after hatching. In the study, *P*-values indicate the confidence level of statistical analyses, with *P* < 0.05 indicating statistically significant differences. ** Represents *P* < 0.01 and *** represents *P* < 0.001.

Alpha diversity analysis primarily assesses the richness and diversity of microbial communities within samples using multiple diversity indices. A higher Shannon index value indicates greater species diversity, whereas a higher Chao index value indicates greater species richness within the sample. The results of alpha diversity analysis indicated significant differences in the Shannon and Chao indices of bacterial communities in the sand of nests before and after hatching (*P* < 0.001; Fig. 3). The Shannon and Chao indices of bacterial communities in the sand of nests before hatching were significantly higher than those after hatching.

**Fig 4:**
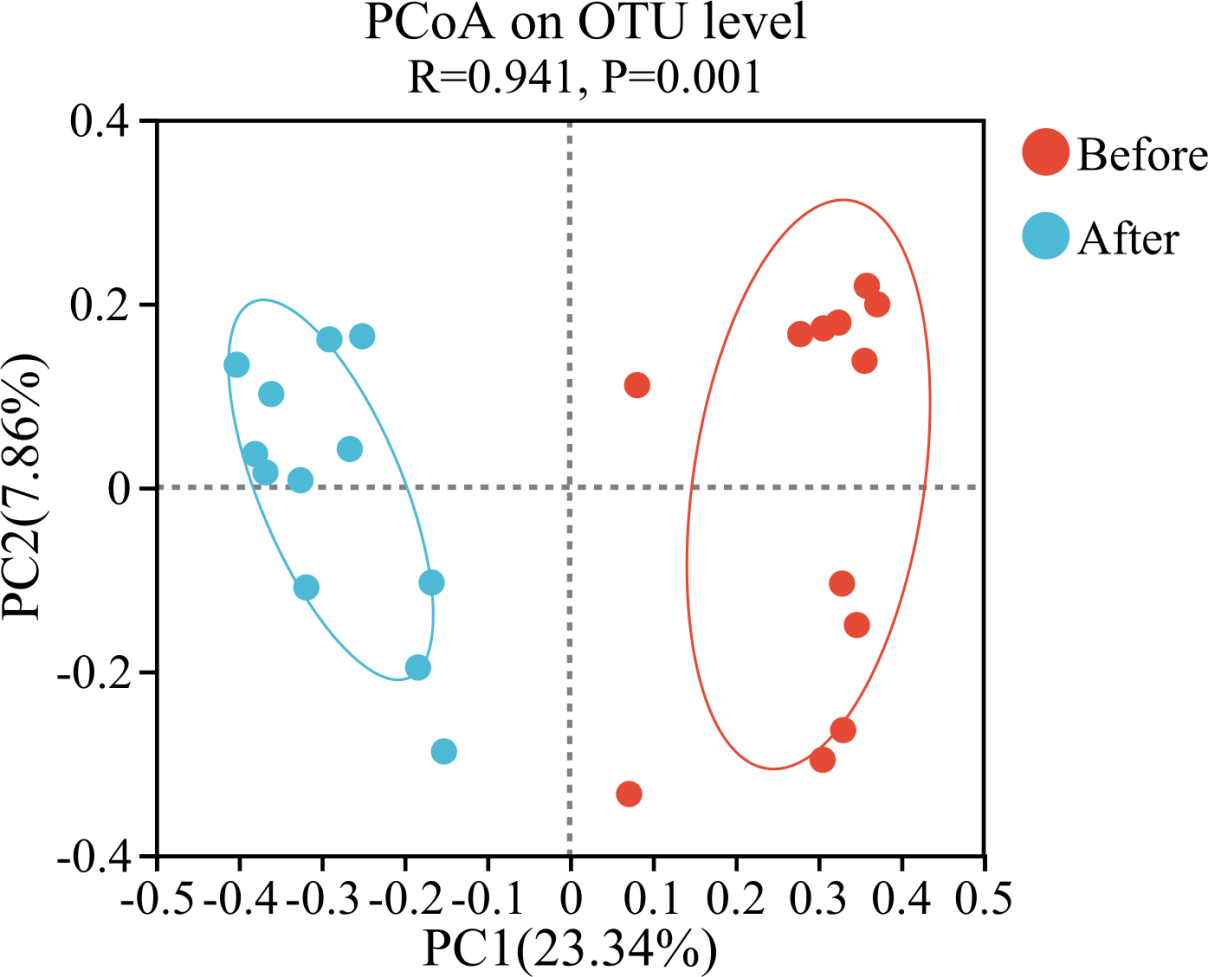
PCoA plot of beta diversity of bacterial communities in the sand of nests before and after hatching.

Principal coordinates analysis (PCoA) of beta diversity was performed using the Bray–Curtis distance algorithm. PCoA results after analysis of similarities (ANOSIM) testing indicated that the bacterial community structures in the sand of nests before and after hatching were clearly separated in the principal coordinates. Two main clusters indicated significant differences between bacterial communities (R = 0.941, *P* = 0.001; Fig. 4).

#### Analysis of microbial community structure composition at the phylum level

**Fig 5:**
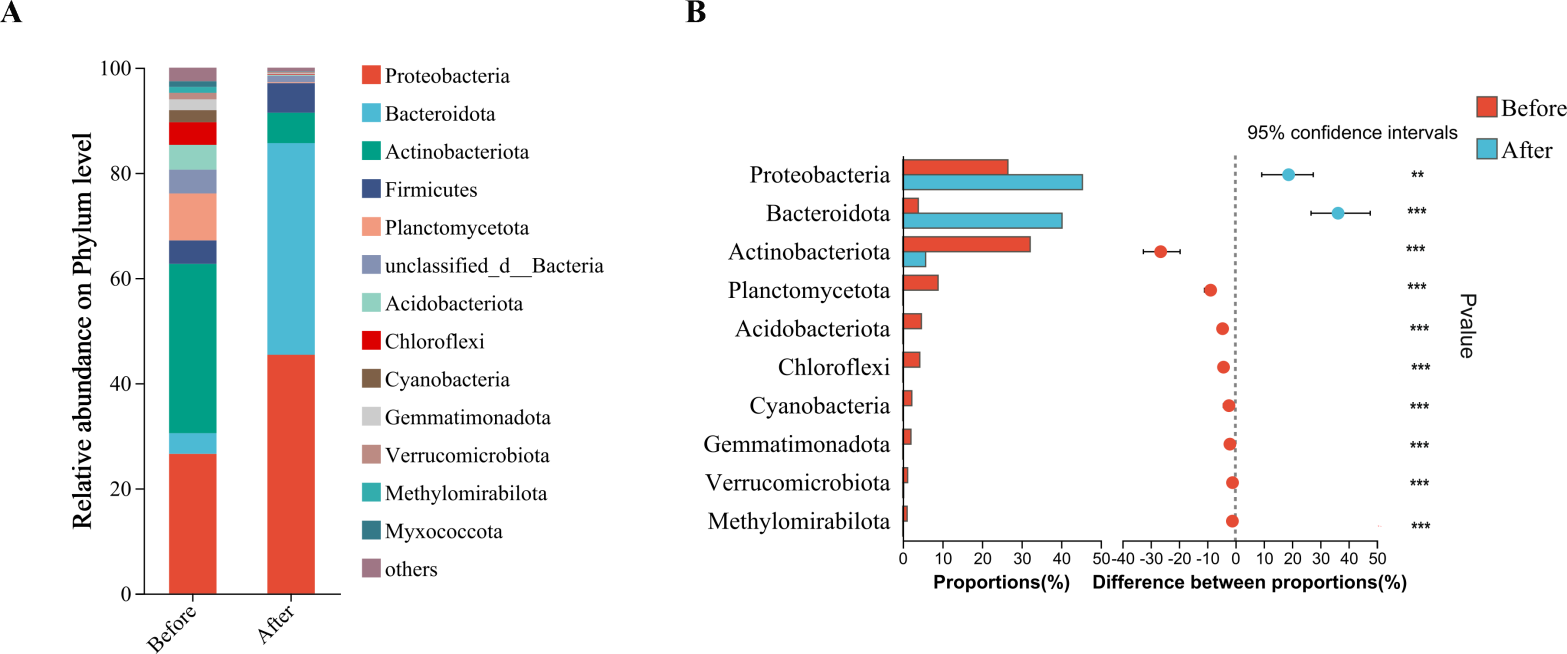
Composition and relative abundance of bacteria at the phylum level in the sand of nests before and after hatching (**A**). Species with significant differences in bacterial composition at the phylum level (**B**). The figure shows only phyla with relative abundances >1% and ranked within the top 10. * Represents *P* < 0.05, ** represents *P* < 0.01, and *** represents *P* < 0.001.

A total of 38 and 32 bacterial phyla were annotated in the sand of nests before and after hatching, respectively. A total of 13 species with relative abundances >1% at the phylum level were plotted as a stacked bar chart (Fig. 5A). The dominant bacterial phyla in the sand of nests before hatching in descending order were Actinobacteriota (32.20%), Proteobacteria (26.57%), Planctomycetota (8.92%), and Acidobacteriota (4.71%). However, the dominant phyla in the sand of nests after hatching in descending order were Proteobacteria (45.40%), Bacteroidota (40.25%), Actinobacteriota (5.78%), and Firmicutes (5.64%).

The top 10 phyla based on relative abundance were selected for inter-group significance testing using the Wilcoxon rank-sum test, which indicated that significant changes occurred in bacterial phyla between the sand of nests before and after hatching (*P* < 0.01; Fig. 5B). The differences were significant in the phyla Proteobacteria, Bacteroidota, Actinobacteriota, Planctomycetota, Acidobacteriota, Gemmatimonadota, Chloroflexi, Cyanobacteria, and Verrucomicrobiota.

#### Analysis of microbial community structure composition at the genus level

**Fig 6:**
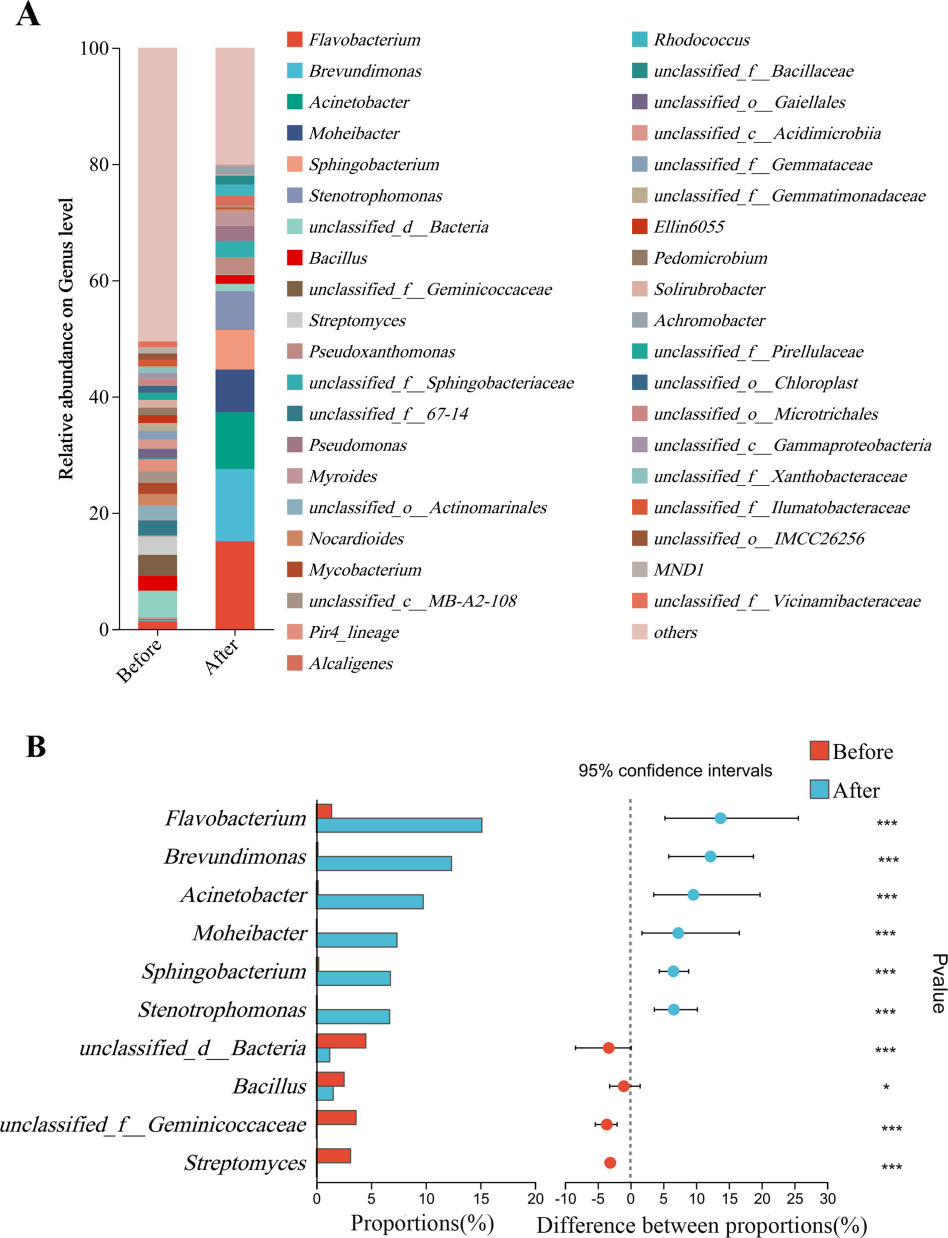
Composition and relative abundance of bacteria at the genus level in the sand of nests before and after hatching (**A**). Species with significant differences in bacterial composition at the genus level (**B**). The figure shows only genera with relative abundances >1% and ranked within the top 10. * Represents *P* < 0.05, ** represents *P* < 0.01, and *** represents *P* < 0.001.

A total of 1,018 annotated OTU genera were identified. A total of 471 and 48 unique genera from the sand of nests before and after hatching were observed, and 499 genera were shared between both categories. A total of 40 bacterial genera with a relative abundance >1% were identified (Fig. 6A). The top five dominant bacterial genera in the sand of nests before hatching were unclassified_d__Bacteria (4.53%), unclassified_f__Geminicoccaceae (3.63%), *Streptomyces* (3.13%), *Bacillus* (2.54%), and *Flavobacterium* (1.4%). However, the top five dominant bacterial genera in the sand of nests after hatching were *Flavobacterium* (15.15%), *Brevundimonas* (12.37%), *Acinetobacter* (9.78%), *Moheibacter* (7.38%), and *Sphingobacterium* (6.78%).

Based on the results of the Wilcoxon rank-sum test, the differences in bacterial genus level in the sand of nests before and after hatching were significant in *Flavobacterium*, *Brevundimonas*, *Acinetobacter*, *Moheibacter*, *Sphingobacterium*, *Bacillus*, *Streptomyces,* and *Stenotrophomonas* (Fig. 6B).

#### LEfSe differential discriminant analysis

**Fig 7:**
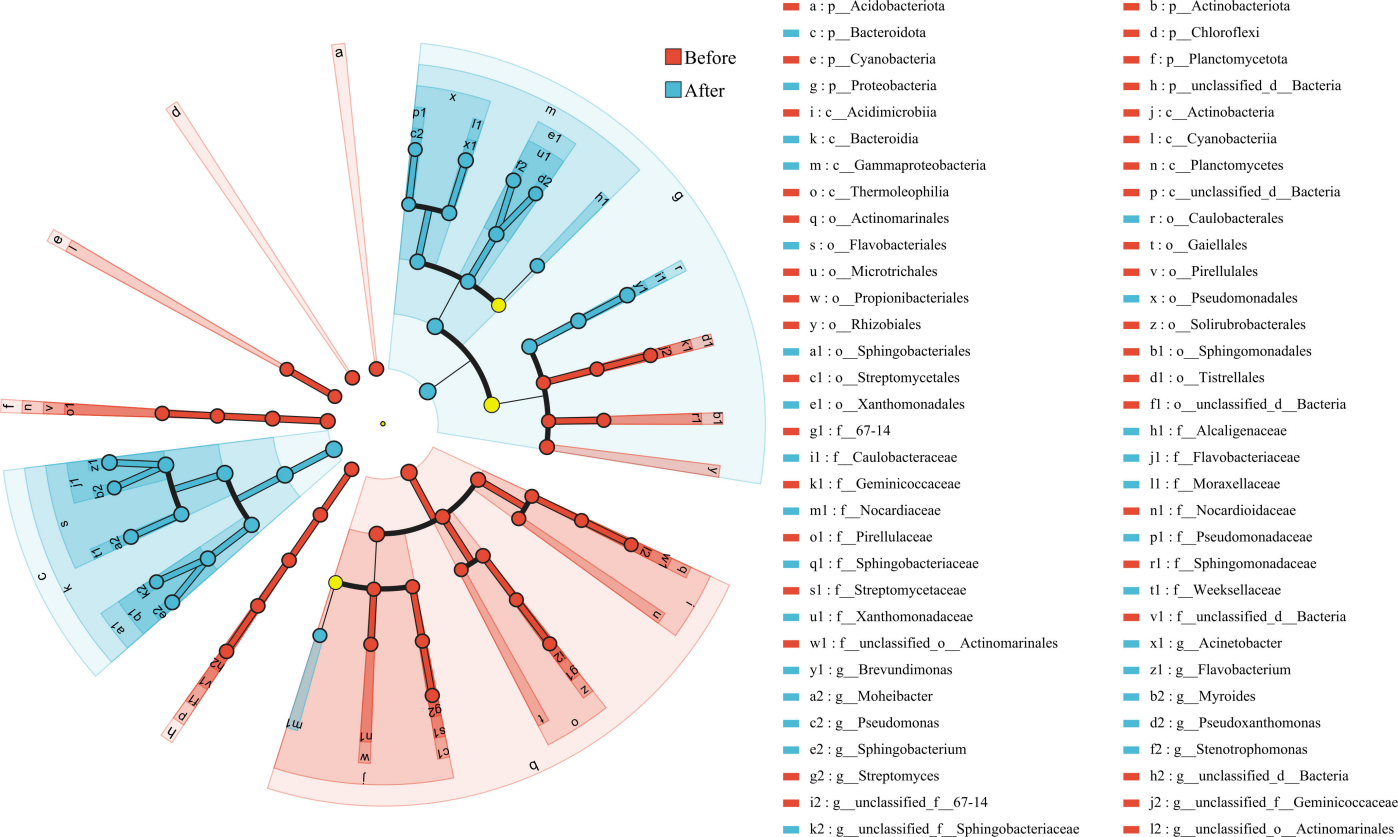
LEfSe analysis of the sand of nests before and after hatching. Displays all taxa with LDA scores >4. The diameter of each circle reflects its abundance. Inside-out circles reflect classification from phylum to genus.

LEfSe multilevel species discriminant analysis evaluated the differences in the relative abundance of bacterial taxa in the sand of nests of green turtles before and after hatching. The dendrogram visually depicts the main differential taxa between groups at various taxonomic levels from phylum to genus. A total of 64 distinctive differential taxa were identified in the sand of nests before and after hatching (Fig. 7), after the linear discriminant analysis (LDA) score threshold was set to 4. Among these, 36 taxa were particularly from the sand of nests before hatching, contributing to most differences in species composition observed between the groups. Additionally, significant differences at the phylum and genus taxonomic levels were observed in the sand samples from the nests before and after hatching across 8 phyla and 15 genera. Actinobacteriota, Planctomycetota, Acidobacteriota, Chloroflexi, and Cyanobacteria were significantly enriched in the sand of nests before hatching. Bacteroidota and Proteobacteria were significantly enriched in the sand of nests after hatching.

### Comparative analysis of microorganisms among hatched eggshells, unhatched egg contents, and gastrointestinal tracts of deceased hatchlings

#### Analysis of alpha and beta diversities

**Fig 8:**
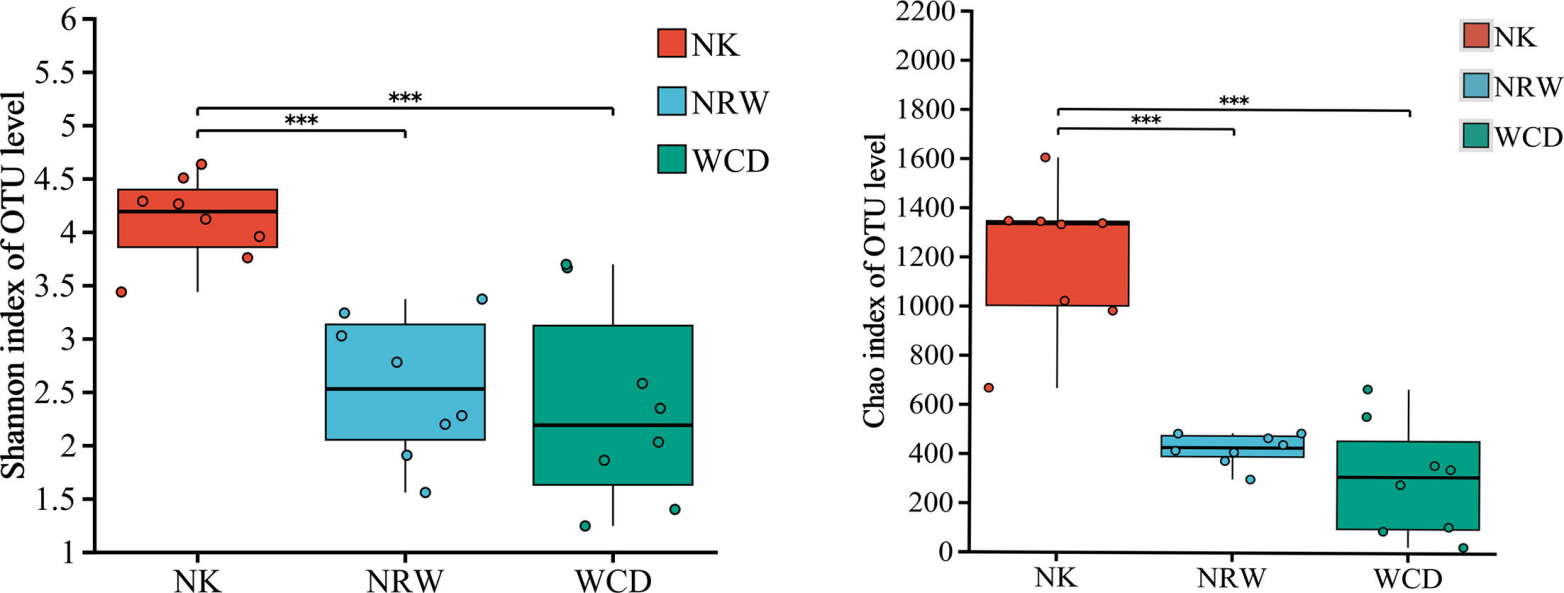
Alpha diversity analysis of bacteria in hatched eggshells (NK), unhatched egg contents (NRW), and gastrointestinal tracts of deceased hatchlings (WCD). * Represents *P* < 0.05, ** represents *P* < 0.01, and *** represents *P* < 0.001.

Results of the alpha diversity index analysis indicated significant differences in the Shannon and Chao indices between the hatched eggshells, unhatched egg contents, and gastrointestinal tracts of deceased hatchlings (*P* < 0.001; Fig. 8). The Shannon and Chao indices of hatched eggshells were significantly higher than those of the unhatched egg contents and the gastrointestinal tracts of deceased hatchlings (*P* < 0.001; Fig. 8); however, no significant differences in the Shannon and Chao indices were observed between the unhatched egg contents and the gastrointestinal tracts of deceased hatchlings (*P* > 0.05; Fig. 8).

**Fig 9:**
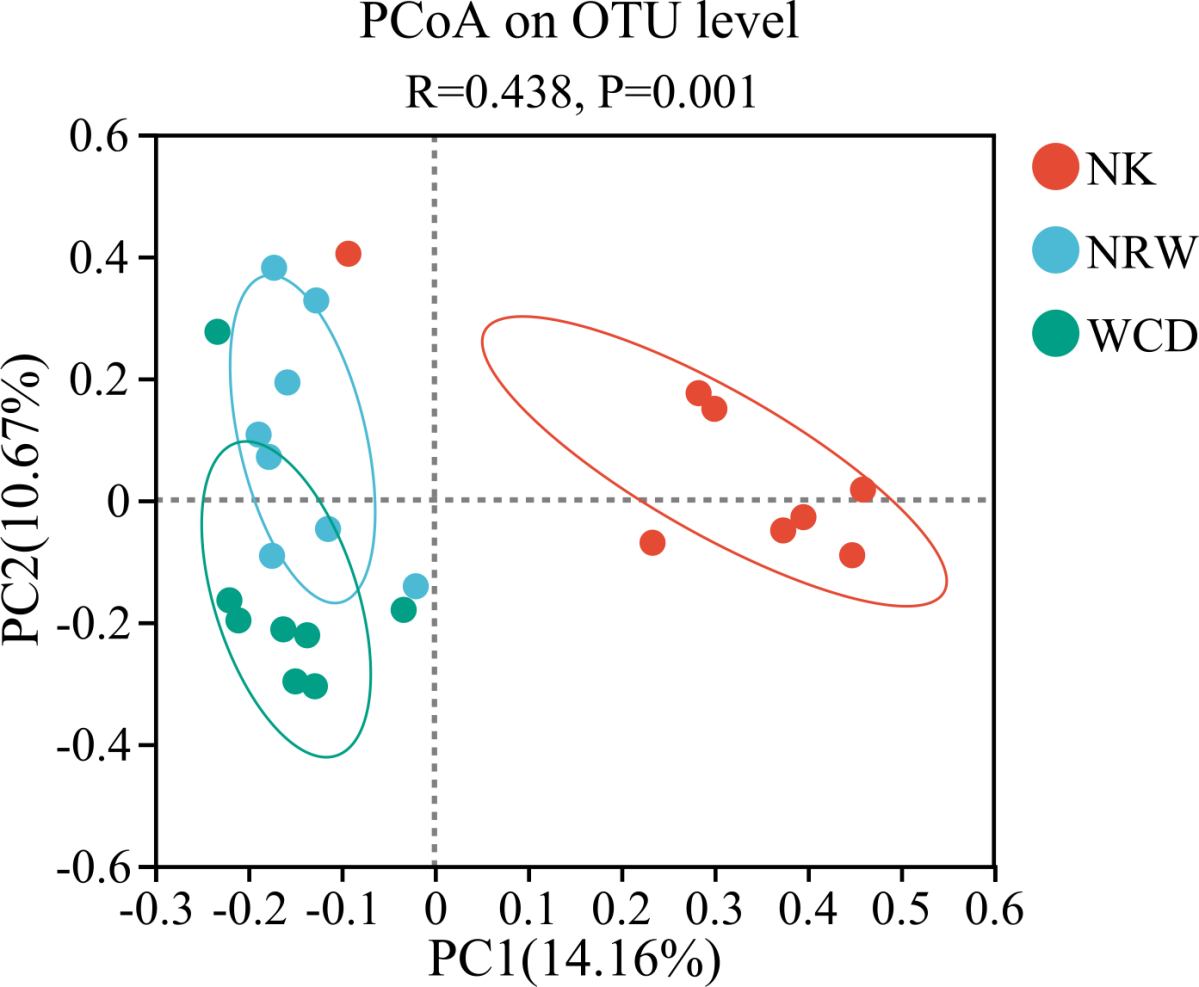
PCoA plot of beta diversity of bacterial communities in hatched eggshells (NK), unhatched egg contents (NRW), and gastrointestinal tracts of deceased hatchlings (WCD).

The results of ANOSIM testing indicated significant differences in the microbial communities between hatched eggshells, unhatched egg contents, and gastrointestinal tracts of deceased hatchlings (*R* = 0.438, *P* = 0.001; Fig. 9).

#### Analysis of microbial community structure composition at the phylum level

**Fig 10:**
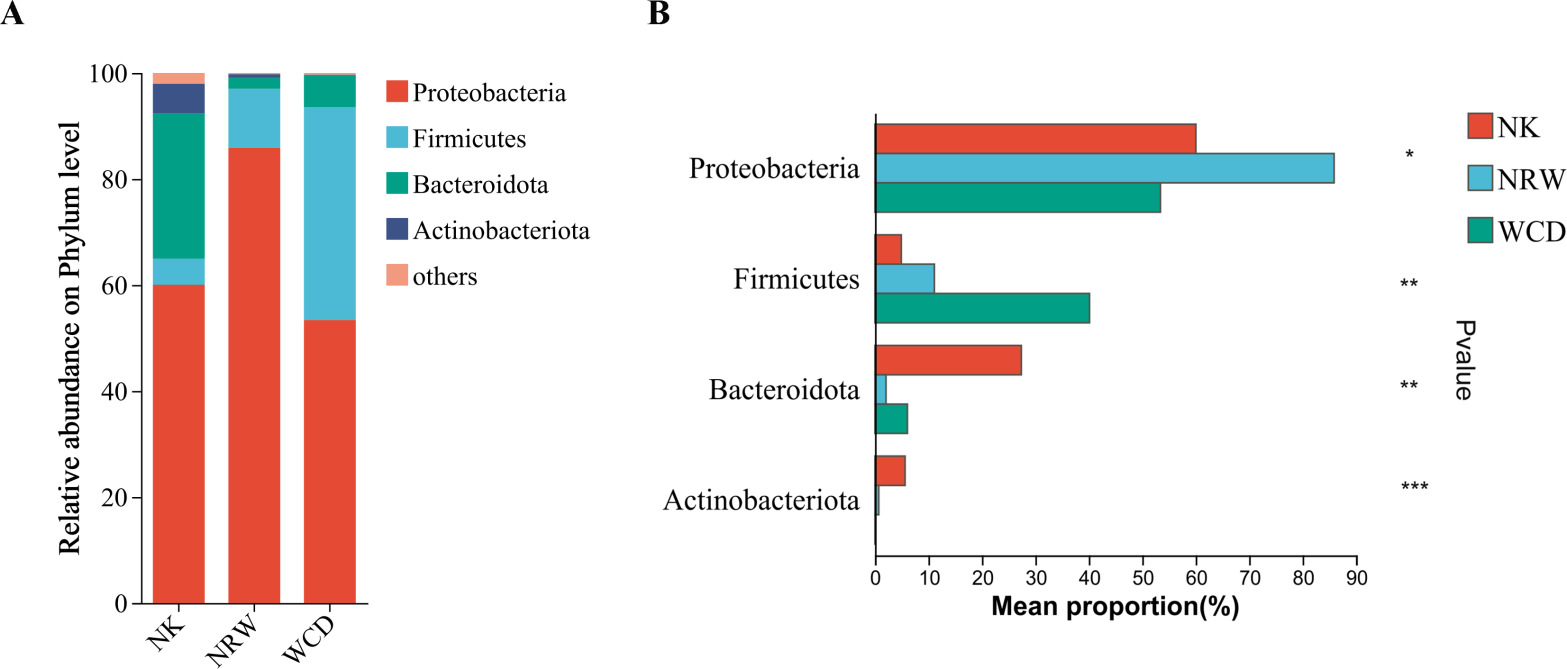
Composition and relative abundance of bacteria at the phylum level in hatched eggshells (NK), unhatched egg contents (NRW), and gastrointestinal tracts of deceased hatchlings (WCD) (**A**). Species with significant differences in bacterial composition at the phylum level (**B**). The figure shows only phyla with relative abundances >1% and ranked within the top 10. * Represents *P* < 0.05, ** represents *P* < 0.01, and *** represents *P* < 0.001.

Hatched eggshells, unhatched egg contents, and gastrointestinal tracts of deceased hatchlings were annotated with 19, 8, and 8 bacterial phyla, respectively. Species with relative abundances >1% were selected for plotting (Fig. 10A). The dominant phyla in the hatched eggshells were Proteobacteria (60.04%), Bacteroidota (27.38%), Actinobacteriota (5.63%), and Firmicutes (4.93%). However, the dominant phyla in the unhatched egg contents were Proteobacteria (85.90%), Firmicutes (11.11%), Bacteroidota (2.07%), and Actinobacteriota (0.71%), and that in the gastrointestinal tracts of deceased hatchlings were Proteobacteria (53.41%), Firmicutes (40.14%), Bacteroidota (6.07%), and Actinobacteriota (0.07%).

Based on the obtained community abundance data, we conducted significance testing of phyla with relative abundance >1% using the Kruskal–Wallis rank-sum test. The results indicated significant differences in the relative abundance of Proteobacteria, Firmicutes, Bacteroidota, and Actinobacteriota among hatched eggshells, unhatched egg contents, and gastrointestinal tracts of deceased hatchlings (Fig. 10B).

#### Analysis of microbial community structure composition at the genus level

**Fig 11:**
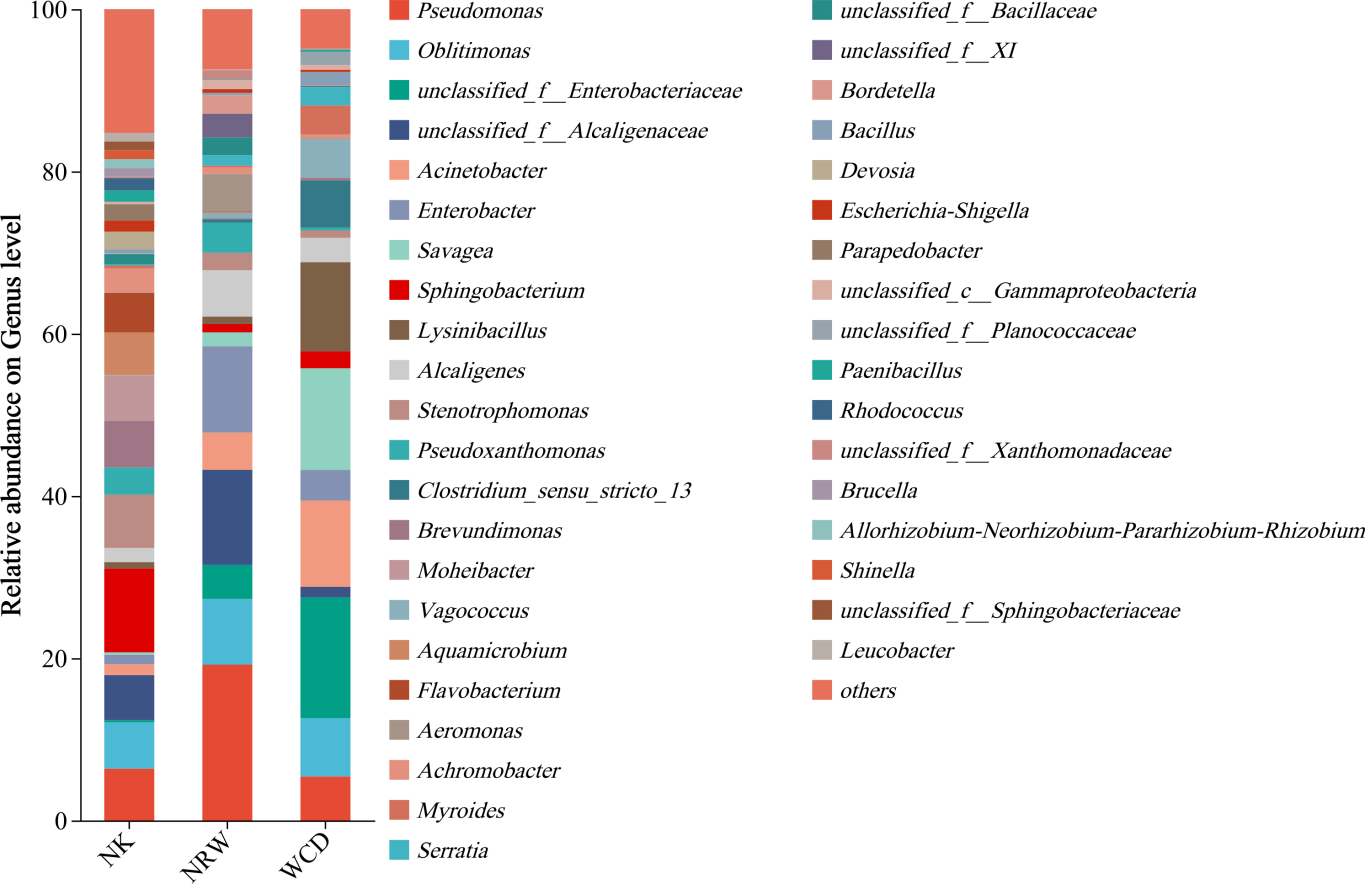
Composition and relative abundance of bacteria at the genus level among hatched eggshells (NK), unhatched egg contents (NRW), and gastrointestinal tracts of deceased hatchlings (WCD).

Based on the OTU taxonomic results, 39 bacterial genera had relative abundances >1% (Fig. 11). Hatched eggshells, unhatched egg contents, and gastrointestinal tracts of deceased hatchlings were annotated with 130, 163, and 121 bacterial genera, respectively. Among these, 188, 18, and 3 unique genera from the hatched eggshells, unhatched egg contents, and gastrointestinal tracts of deceased hatchlings were observed, and 100 genera were common to all three categories. The dominant bacterial genera in the hatched eggshells were *Sphingobacterium* (10.34%), *Stenotrophomonas* (6.56%), *Pseudomonas* (6.40%), and *Brevundimonas* (5.73%). However, the dominant bacterial genera in the unhatched egg contents were *Pseudomonas* (19.21%), *Enterobacter* (10.64%), *Oblitimonas* (8.10%), and *Alcaligenes* (5.71%), and that in the gastrointestinal tracts of deceased hatchlings were *Savagea* (12.53%), *Lysinibacillus* (11.01%), *Acinetobacter* (10.64%), and *Oblitimonas* (7.18%).

**Fig 12:**
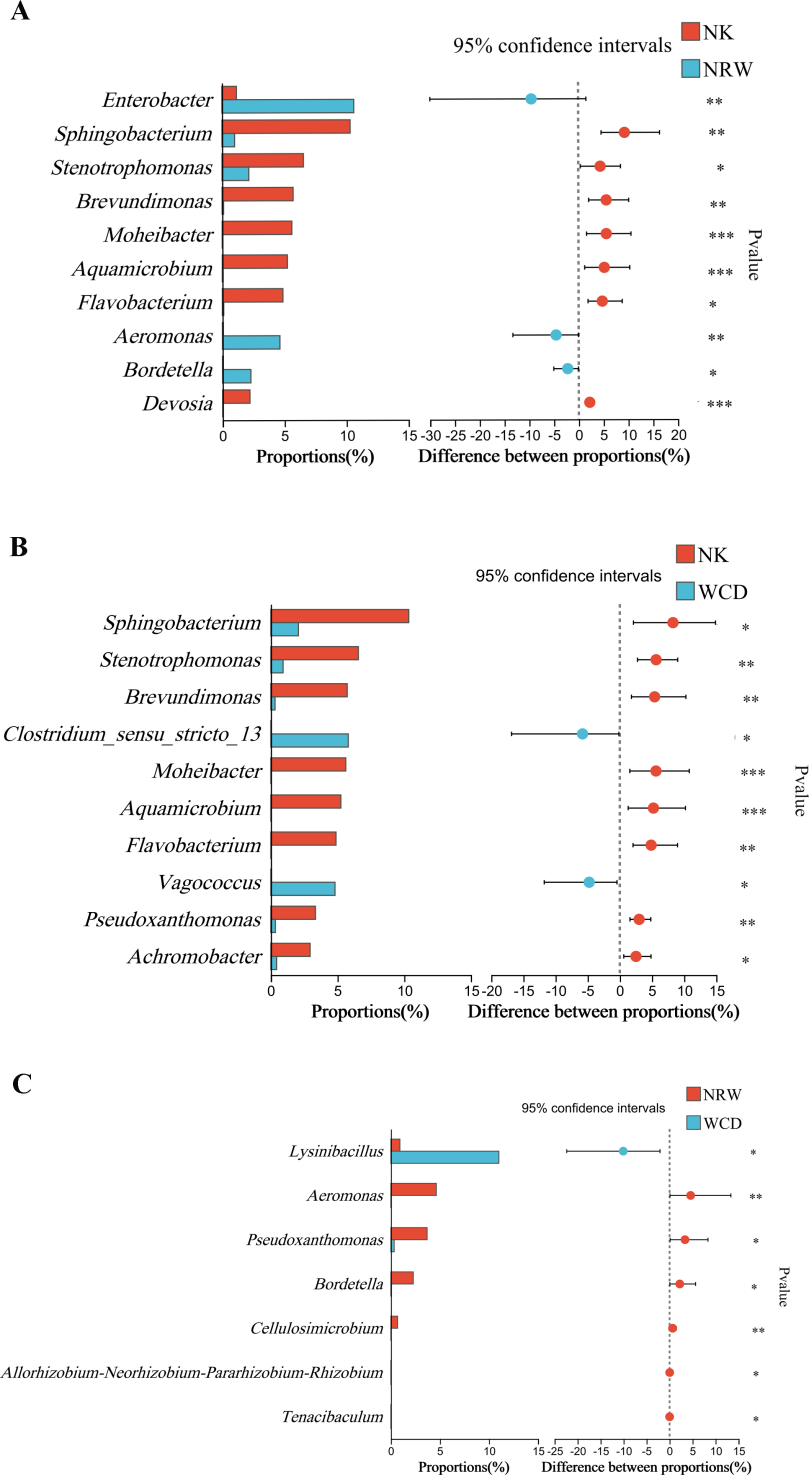
Species with significant differences in bacterial composition at the genus level: hatched eggshells and unhatched egg contents (A), hatched eggshells and the gastrointestinal tracts of deceased hatchlings (B), unhatched egg contents and the gastrointestinal tracts of deceased hatchlings (C). The figure shows only genera with relative abundances >1% and ranked within the top 10. * Represents *P* < 0.05, ** represents *P* < 0.01, and *** represents *P* < 0.001.

Wilcoxon rank-sum test was performed on the top 10 bacterial genera based on relative abundance rankings (Fig. 12). The differences in the relative abundance between hatched eggshells and unhatched egg contents at the genus level were significant in *Enterobacter*, *Aeromonas*, *Bordetella*, *Sphingobacterium*, *Stenotrophomonas*, *Brevundimonas*, *Moheibacter*, *Aquamicrobium*, *Flavobacterium,* and *Devosia* (Fig. 12A). The differences in the relative abundance between hatched eggshells and the gastrointestinal tracts of deceased hatchlings at the genus level were significant in *Clostridium_sensu_stricto_13*, *Vagococcus*, *Sphingobacterium*, *Stenotrophomonas*, *Brevundimonas*, *Moheibacter*, *Aquamicrobium*, *Flavobacterium*, *Pseudoxanthomonas,* and *Achromobacter* (Fig. 12B). The differences in the relative abundance between unhatched egg contents and the gastrointestinal tracts of deceased hatchlings at the genus level were significant in *Lysinibacillus*, *Aeromonas*, *Pseudoxanthomonas*, *Tenacibaculum*, *Bordetella, Cellulosimicrobium,* and *Allorhizobium-Neorhizobium-Pararhizobium-Rhizobium* (Fig. 12C).

#### LEfSe differential discriminant analysis

**Fig 13:**
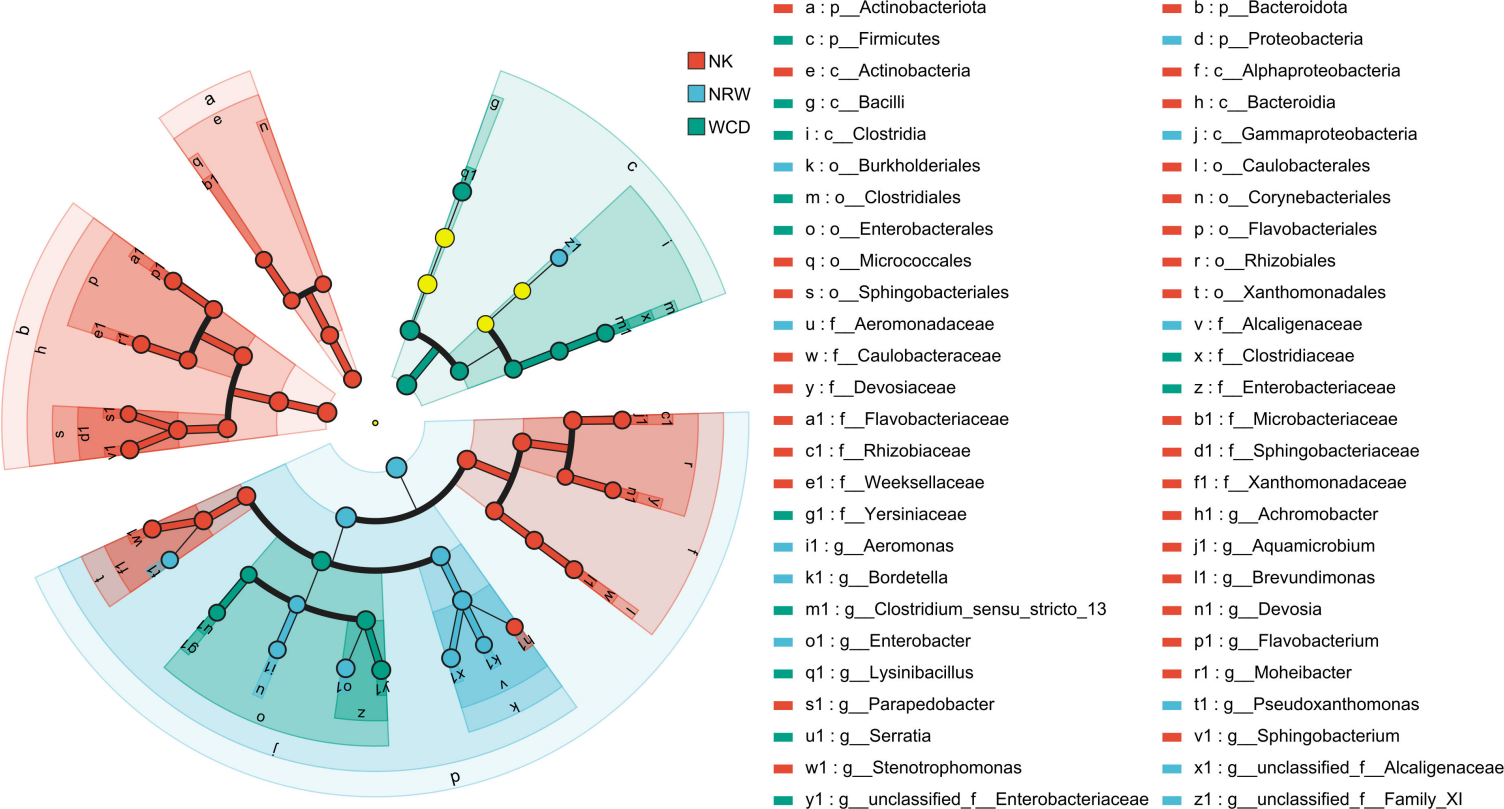
LEfSe analysis of hatched eggshells (NK), unhatched egg contents (NRW), and gastrointestinal tracts of deceased hatchlings (WCD), displays all taxa with LDA scores >4. The diameter of each circle reflects its abundance. Inside-out circles reflect classification from phylum to genus.

A total of 52 taxa exhibited significant differential abundance among hatched eggshells, unhatched egg contents, and gastrointestinal tracts of deceased hatchlings (Fig. 13) after LEfSe analysis, with an LDA score threshold set to 4. Additionally, significant differences at the phylum and genus taxonomic levels were observed among the three categories across 4 phyla and 19 genus levels. Bacteroidota and Actinobacteriota were significantly enriched in hatched eggshells, Proteobacteria were significantly enriched in unhatched egg contents, and Firmicutes were significantly enriched in the gastrointestinal tracts of deceased hatchlings.

## DISCUSSION

### Comparative analysis of bacteria in the sand of nests before and after hatching

In this study, significant differences were observed in both alpha and beta diversities of bacterial communities in the sand of nests before and after hatching (*P* < 0.001; Fig. 3 and 4), which indicated that the hatching of green turtle eggs alters the original microbial community structure characteristics of the sand of nests. The sand of nests before hatching had a richer diversity of microbial taxa than the sand of nests after hatching. Our findings were consistent with the findings of previous studies (21). Previous researches speculated that the following factors may be involved: (i) environmental fluctuations, such as changes in nest temperature and humidity at the nesting grounds during the hatching period, (ii) mucous secretion from the cloaca of female sea turtles during egg laying (22); these secretions provided a relatively warm and humid environment, along with potential nutrients, which promoted the growth of microbes within the nests (23); however, these secretions may also contain antimicrobial properties that could restrict the growth of certain microbes (24, 25).

The relative abundance of Proteobacteria and Actinobacteria at the phylum level significantly increased in the sand of nests after hatching. Both phyla are among the most common in marine and sandy environments and are involved in functions such as the enrichment of acidic amino acids, hydrolysis of cellulose, and mineralization of organic compounds (26). Conversely, the relative abundances of Actinobacteriota, Planctomycetota, Acidobacteriota, Chloroflexi, Cyanobacteria, Gemmatimonadota, and Verrucomicrobiota significantly decreased in the sand of nests after hatching, indicating that these phyla may have been suppressed during the hatching process of green turtles. However, sea turtle eggs hatch in the natural environment, where there are many uncontrollable factors, making it difficult to determine the specific influencing factors.

The relative abundance of *Bacillus* and *Streptomyces* significantly decreased in the sand of nests after hatching than in the sand of nests before hatching. In contrast, the relative abundance of *Flavobacterium*, *Brevundimonas*, *Acinetobacter*, *Moheibacter*, *Sphingobacterium,* and *Stenotrophomonas* significantly increased in the sand of nests after hatching. *Streptomyces* was the dominant genus in the sand of nests before hatching; many *streptomycetes* can produce a variety of secondary metabolites, including various enzymes for the degradation of substances and antibiotics (27). Sarmiento-Ramírez et al. (11) speculated that *Streptomyces* species may possess antibacterial and antifungal activities, potentially inhibiting the mycelial growth of pathogens such as *Fusarium falciforme*, which are associated with hatchling failure in sea turtles, thereby protecting egg development. The genera *Flavobacterium*, *Acinetobacter,* and *Stenotrophomonas* were the dominant genera in the sand of nests after hatching. Notably, bacteria from the *Flavobacterium* and *Acinetobacter* genera are predominantly opportunistic pathogens that can cause infections; these genera are widely distributed in nature and are commonly found in soil and marine environments. The genus *Acinetobacter* is associated with human health and is recognized as a pathogen responsible for significant public health issues (28, 29). Immunocompromised patients are susceptible to infections, including meningitis, pneumonia, endocarditis, bacteremia, and skin and urinary tract infections (30, 31). At the same time, the excessive proliferation of these pathogenic bacteria may destroy the original microbial balance in the nest, inhibit other beneficial bacteria, and indirectly affect the quality of subsequent hatched turtle eggs here.

In this study, potentially pathogenic genera significantly increased in the sand of nests after hatching. Although the presence of pathogenic bacteria may be the cause of embryo death, it cannot ultimately be proven that way, and not all bacteria that infect turtle eggs are necessarily pathogenic to turtles. Pathogens only affect organisms under suitable conditions. The nests of sea turtles provide an ideal environment for microbial growth, with hatched eggshells, unhatched eggs, and deceased embryos serving as excellent growth substrates for microorganisms. Excessive microbial growth can further alter the microenvironment, including temperature, humidity, and oxygen levels within the nest, facilitating the proliferation of pathogens and their invasion into turtle eggs. Additionally, proximity between nests facilitates the cross-nest transmission of pathogenic bacteria, thereby expanding the scope of infection (14, 32). Over a long period of accumulation, the presence of these pathogens may disrupt the ecological balance of the microbial communities in nesting grounds and increase the susceptibility of sea turtle eggs, posing a threat to the hatching of sea turtle eggs and hatchling survival, thereby representing a potential safety risk. Further research is needed to understand the specific mechanisms underlying these differences.

### Comparative analysis of bacteria among hatched eggshells, unhatched egg contents, and gastrointestinal tracts of deceased hatchlings

In this study, alpha diversity analysis based on the Shannon and Chao indices revealed that the bacterial diversity and abundance of hatched eggshells was significantly higher than those of unhatched egg contents and the gastrointestinal tracts of deceased hatchling. Beta diversity analysis indicated significant differences in bacterial community composition between hatched eggshells, unhatched egg contents, and gastrointestinal tracts of deceased hatchlings. The predominant bacterial phyla in the hatched eggshells were Proteobacteria and Firmicutes, consistent with previous studies (11, 19). The predominant genus in the hatched eggshells was *Sphingobacterium*, which is widely distributed in nature, capable of adapting to various complex extreme environments, has complex and diverse metabolic types, and extremely strong adaptability, and rarely causes infections in humans and animals.

*Pseudomonas* was the predominant bacteria genus in the unhatched egg contents, which is consistent with the findings of McMaken et al. (6). The genus *Pseudomonas* is a Gram-negative bacterium capable of causing “melioidosis” in humans and animals, potentially leading to sepsis or shock, and is a pathogen responsible for illness in captive sea turtles (9). Craven et al. (33) and Capri et al. (15) detected the genus *Pseudomonas* in unhatched egg contents and soil of nests and speculated that this may be related to the failure of sea turtles to hatch eggs. Al-Bahry et al. (9, 34) conducted a bacterial contamination analysis on the eggs of green turtles and discovered that 42% of the eggs were contaminated with pathogenic bacteria, with the genus *Pseudomonas* being the most common (30.3%), followed by genera such as *Enterobacter* (14.3%). The relative abundance of *Enterobacter*, an opportunistic pathogen in plants, animals, and humans, was significantly higher in unhatched egg contents than in hatched eggshells. However, the pathogenic mechanisms of this bacterium remain unclear. Additionally, the relative abundance of *Aeromonas* in unhatched egg contents was significantly higher than that in the hatched eggshells. The genus *Aeromonas* can penetrate the eggshell and infect the egg, using the nutrients in the egg to proliferate (35). *Aeromonas* is a ubiquitous bacterium found in both terrestrial and aquatic environments, considered a significant pathogen of fish and other cold-blooded animals, and can also cause infections and complications in both immunocompetent and immunocompromised individuals (36).

*Acinetobacter* was the third most dominant genus in the gastrointestinal tracts of deceased hatchlings that can cause human disease, with a relative abundance of 10.64%. We discovered that *Acinetobacter* was a dominant genus in the sand of nests before and after hatching; however, the impact of *Acinetobacter* on the hatching of eggs of sea turtles has not yet been reported. The relative abundance of *Lysinibacillus* in the gastrointestinal tracts of deceased hatchlings was significantly higher than that in the hatched eggshells and unhatched egg contents. The genus *Lysinibacillus* is a Gram-positive bacterium belonging to the Firmicutes, and species within this genus were originally discovered for their insecticidal properties (37); however, the effects of *Lysinibacillus* on sea turtles have not been reported.

The nest sand has a great influence on the microbial composition of sea turtle eggs (19). Approximately 75% of the bacteria isolated from sea turtle eggs can play a pathogenic role; it has also been detected in other animals (33). Gambino et al. (38) have detected pathogens that may affect hatching bacterial strains belonging to the genera *Aeromonas* and *Citrobacter* in eggs and dead hatchlings from six loggerhead sea turtle nests located on the Sicilian coast. Many studies have proved that fungal species belonging to the genus *Fusarium* are the main cause of the failure of sea turtle egg hatching (39–41), but the species of bacteria have rarely been specifically verified. In this study, the unhatched egg contents had a larger amount of opportunistic pathogens than the hatched eggshells and the gastrointestinal tracts of deceased hatchlings. A higher abundance of *Pseudomonas* was present in unhatched eggs (6, 34), and we speculate that this may be related to the failure of sea turtles to hatch eggs. Therefore, further research is needed to explain this finding.

In conclusion, it is suggested that the nesting grounds should establish a long-term monitoring mechanism of microbial diversity, regularly evaluate the microbial community structure and pathogen abundance of the nesting grounds, and take timely measures to address the problems found.

### Conclusion

Microorganisms in the nesting environment influence the embryo development of sea turtles; therefore, gaining a comprehensive understanding of the microecological environment of the nests of green turtles is crucial. We compared the bacterial community composition of the sand of nests before and after hatching, and between hatched eggshells, unhatched egg contents, and gastrointestinal tracts of deceased hatchlings, explored the impact of sea turtle reproduction and hatching on nests, and revealed the types of potential pathogenic bacteria present in the nests of green turtles. Our results can provide scientific support for the planning, environmental management, protection, and restoration of the nesting grounds in the Xisha Islands. Next research should identify which bacteria may have potential pathogenic effects on the eggs of sea turtles, so as to monitor the nesting grounds in a targeted manner.

## Data Availability

These sequence data have been submitted to the NCBI Sequence Read Archive (SRA) under accession number PRJNA1199617.
